# Stability and change in male fertility patterns by cognitive ability across 32 birth cohorts

**DOI:** 10.1098/rsbl.2023.0172

**Published:** 2023-06-28

**Authors:** Bernt Bratsberg, Ole Rogeberg

**Affiliations:** Ragnar Frisch Centre for Economic Research, Oslo, Norway

**Keywords:** fertility, cognitive ability, dysgenics

## Abstract

The relationship between cognitive ability (CA) and childbearing remains unsettled. Using Norwegian administrative registers with population coverage, we study how male lifetime fertility patterns differ across cognitive score groups, and how these changed across the 1950–1981 birth cohorts, covering a period characterized by rapid social and economic change. The analyses reveal systematic differences in fertility and fertility timing across CA groups, with high scoring males having delayed but ultimately higher fertility than lower scoring males. This pattern remains stable over time despite strong trends towards delayed and reduced fertility. The overall positive relationship between CA and fertility is primarily driven by high rates of childlessness in the lowest scoring group, with low-scoring males showing higher rates of parity progression at higher parities.

## 1. Introduction

Concerns over dysgenic fertility continue to be raised in the academic discourse. Indeed, a recent systematic review concluded that a negative association between cognitive ability (CA) and fertility in modern Western societies strengthened over the course of the twentieth century, attributing the development to social and economic progress that disproportionately raised the reproduction rates in the lower part of the ability distribution [1]. The long-term consequences are held to be dire, with the review stating that ‘*the decline of a heritable trait so crucial to health, occupational, and socio-behavioral outcomes yields substantial ramifications at both the individual and national level [… . ] Should this trend continue unabated, coupled with continuing overpopulation, there is a risk it will eventually trigger economic stagnation and decline, and civil instability*’ (p. 116).

The hypothesis that social welfare policies raise the relative fertility of low-ability parents, contrasts with the results of a recent study from Sweden. The study used administrative data with full population coverage to assess the CA–fertility gradient for the 1951–1967 male birth cohorts [2]. Although Sweden is known for its strong welfare state, the data showed a strong positive relationship between CA scores from military conscription, typically at age 18–20, and lifetime fertility. The relationship may also differ across contexts: a study from Iceland reported negative selection on genes associated with education that would imply a 0.3 IQ point decline per decade [3], while UK biobank data indicate negative selection on polygenic scores for CA and educational attainment [4].

In this paper, we examine the case of Norway. Using administrative register data, we extend the analysis to cover more than 30 birth cohorts. This enables us to examine in detail how the association between male CA and fertility changed across a period of extensive societal change: significant expansions of the educational system, rising female participation in the labour force, and the increasing availability of contraception and legal abortion. The period also saw rapid and substantial shifts in overall fertility patterns, with overall male fertility (measured at age 40) declining from 1.9 to 1.5 as the average age of fathers increased by 3 years (electronic supplementary material, figure S2). The causes of this rapid decline remain unclear [5], but by the dysgenic hypothesis we would expect a cognitive gradient in fertility postponements from rising education levels and fertility planning, resulting in more dysgenic fertility.

Our analysis covers 953 692 Norwegian-born males across the 1950–1981 birth cohorts, using stanine scores from military conscription tests and fertility data from national administrative registers with full population coverage. Conscription was voluntary and rare for women, precluding further analysis. The registers cover all births from 1960 through 2021, totaling 1 706 438 observed births registered to our paternal cohorts. The conscription test data cover the majority of males in every birth cohort, with coverage increasing from 72% to 86% across the 1950–1960 cohorts and exceeding 90% for all but two cohorts born after 1961 (electronic supplementary material, figure S1).

We employ two statistical models to describe nuanced fertility patterns within these data. The first model examines average fertility at different ages for men with different CA stanine scores born in different years, using a hierarchical structure with shrinkage to smooth patterns. The second model assesses individual fertility histories (up to age 50) across cells defined by the father's birth cohort and stanine score, exploring differences and trends in childlessness rates and parity progression probabilities. Finally, we reproduce key patterns uncovered by these analyses using the ‘raw’ register data to ensure that results are not the artefacts of statistical modelling.

## 2. Material and methods

The data for this study were obtained from Norwegian administrative registers, provided by Statistics Norway [6]. Individual pseudonymous identifiers were used to link information from the population registry, including births and registered parents, to stanine scores from military conscription testing (typically at age 18–19) for males born in 1950 or later. The CA data, which have been extensively used and described in prior research [7–9], comprise an aggregate integer score reflecting sub-scores on three timed tests: vocabulary (54 items), arithmetic (30 items) and figures (‘Raven-like’ matrices, 36 items). While test items remained unchanged throughout, a renorming of the test in 1980 shifted scores by one stanine, reducing the number of time-invariant score categories to 8.^1^

Descriptive statistical models are used to smooth random variation in the data. Model 1 estimates the average probability of childbirths within cells defined by the father's birth cohort, age and stanine score using a logit specification. The model parameters are divided into two blocks, with the first block containing coefficients for first-order indices (birth cohort, age, calendar year and stanine score), and the second block containing second-order or interaction indices representing all combinations of the stanine score levels with each of the first-order indices. Both blocks of coefficients are assigned zero-centred normal distribution priors, with hyperparameters controlling the scales of the priors. See electronic supplementary material for further details.

In brief, the model assumes that there are underlying patterns of similarities across cells (e.g. fertility being higher at certain ages), which can be exploited to better predict fertility propensity within each individual cell. The hierarchical structure reduces overfitting to noise by inducing shrinkage in the estimates.

Model 2 estimates the conditional probability of having additional children before the age of 50, based on the number of previous children (0, at least 1, 2, 3 or 4). This is treated as analogous to a duration model, where all males in a birth cohort are at risk of having a child, and only those who have a child are at risk of having a second child, and so on. Children of parity >5 are excluded from the parity progression analysis.

The probabilities are specified using a logit model, with parameters again divided into two blocks. The first block (first-order) consists of indices for birth cohort, stanine score and past-parity, while the second block (second-order or interactions) includes indices for all two-way interactions between the first-order indices (e.g. cohort × stanine, cohort × parity, stanine × parity). The hyperparameters for the second-order block are set more conservatively than those for the first-order block to reduce volatility and increase smoothing. For further details and model code, see the electronic supplementary material (section D).

Both models are specified and estimated using the Stan programming language for probabilistic modelling [10]. See electronic supplementary material (section D) for model code.

## 3. Results

A summary measure of the CA–fertility association is the difference between the child-weighted and the unweighted mean CA score of the parent generation.^2^ This difference indicates the generational shift in IQ scores if each child were to inherit their father's stanine score. For 22 cohorts observed to age 50 (1950–1971), comprising 572 029 scored potential fathers with 1 104 594 children, the child-weighted and unweighted stanine means are 5.045 and 4.982, respectively. The difference of 0.064 stanines, equivalent to IQ point increases of approximately 0.5 over one generation or about 0.17 IQ point per decade, implies that the observed fertility across the full sample was weighted towards those with above-average CA scores. Note that this is only intended as a summary indicator of the cognitive fertility gradient for men. It should not be viewed as an estimate of generational IQ change, which will also be affected by the cognitive fertility gradient for women as well as the heritability of CA.

**Figure 1.**
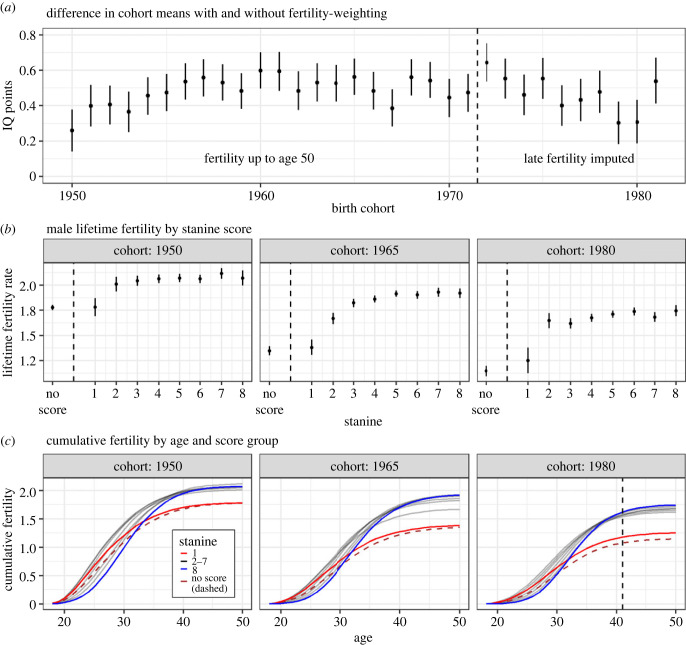
Male fertility patterns by cognitive ability (CA), birth cohort, and age. (*a*) The difference between weighted and unweighted average birth cohort IQ scores, with weights given by cohort fertility. In panels (*a*) and (*c*), the vertical dashed line separates cohorts with fully observed fertility from those with imputed fertility at high ages. The bars are 95% credibility intervals. (*b*) The expected lifetime fertility rate by CA score group for men in three cohorts, along with 95% credibility intervals. (*c*) The cumulative fertility rate of each CA stanine across the lifespan, with the lowest and highest stanines emphasized, and with the vertical dashed line for the 1980 cohort separating the model results for the ages with observed and imputed fertility. The unscored group is shown as a dashed line. Cohorts vary in size: 32 792 (1950), 29 949 (1965), 24 529 (1980).

Estimated at the birth cohort level, this summary measure of the CA–fertility association shows no long-term trend, varying around the overall sample mean (figure 1*a*). This indicates a stable, overall positive association between CA and male fertility. Although fertility is declining monotonically over birth cohorts, the decline is of similar magnitude across stanine groups (figure 1*b*). Notably, the lowest-scoring males consistently have markedly lower fertility within each cohort.

Distinct CA-specific age profiles of fertility emerge from the data. Low-scoring males persistently have the highest fertility at young ages, while higher-scoring males begin later but ultimately end up with higher fertility (figure 1*c*). There has been a general shift towards delayed and reduced fertility for all CA groups across the studied cohorts.

Note that figure 1*a*, by excluding unscored males, likely yields conservative estimates, as unscored males tend to have scores in the lower part of the CA distribution [9] with fertility in line with the lowest scoring males (figure 1*b*). Their inclusion would consequently further increase the gap between the child-weighted and unweighted means.

**Figure 2.**
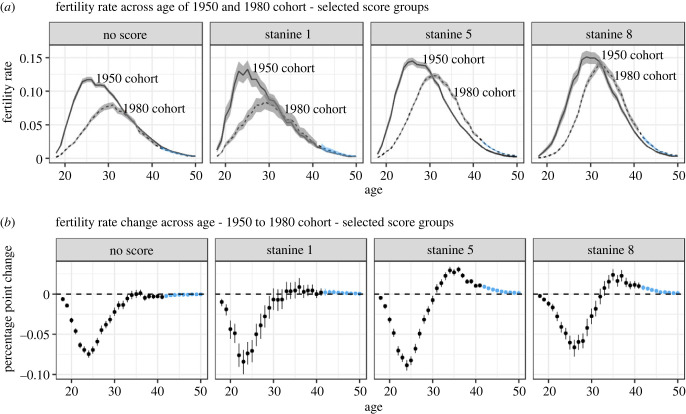
Change in fertility by age for three cognitive ability (CA) stanines and unscored men, 1950–1980. Imputed values for yet unobserved fertility marked in blue. (*a*) The age-specific fertility rate with 95% credibility intervals (shaded areas) for two birth cohorts of three CA stanines using a cohort's number of males alive at age 18 as the denominator. (*b*) The estimated age-specific change in fertility from 1950 to 1980 for the same CA stanines, with 95% credibility intervals depicted by vertical lines.

To assess how fertility age profiles have shifted over time for different score groups, we compare the fertility–age profiles of the 1950 and 1980 birth cohorts for three different score groups (figure 2*a*). This reveals a stark decline in fertility at ages below 30 across all CA groups, partly offset in the higher CA groups by an increase in fertility after the age of 30 (figure 2*b*).^3^ Repeating the analysis to assess cohort shifts across shorter time spans finds large shifts between 1950–1960, smaller shifts in the same direction 1960–1970, and a new pattern 1970–1980 where fertility grew increasingly concentrated around the early thirties with reduced fertility at both lower and higher ages (electronic supplementary material, figure S7).

Using model 2 to estimate parity progression rates as a function of birth year and stanine score, we find that the main driver of the CA–fertility gradient is a higher rate of childlessness among males in the lowest-scoring groups (electronic supplementary material, figure S8*a*). Conditional on having a child (i.e. of parity 1), the probability of having an additional child increases with stanine score (electronic supplementary material, figure S8*b*). For higher parity progressions, the gradient is reversed, with low-scoring males more likely to have additional children. Comparing trends across all cohorts finds childlessness rates almost doubling, with larger absolute changes for low-scoring males. Changes at later parity progressions are more moderate (electronic supplementary material, figure S9).

## 4. Discussion

Norwegian data from 32 male birth cohorts shows a stable positive CA–fertility gradient across a period of rapid social changes: increased female education and labour force participation, oral contraceptive availability, legal abortion, extended maternity leave, subsidized childcare access and shifts in family structure as divorce rates quadrupled [7]. This strengthening of the welfare state was not associated with a dysgenic shift in male fertility.

Four key patterns emerged from the data. First, fertility and fertility timing differed systematically across CA score groups, with high-scoring males having delayed yet ultimately higher fertility than low-scoring males. Second, this pattern persisted across all cohorts, despite large cohort-level shifts towards delayed and reduced fertility (electronic supplementary material, figure S2). The changes were large (e.g. a decline of seven percentage points in the probability of having a child at age 26) but occurred across stanine groups (electronic supplementary material, figures S3–S5), with stronger fertility reductions in low-scoring groups (electronic supplementary material, figure S6). Third, while the shift towards delayed fertility seems to have stopped, fertility patterns have not reversed: births at ages below 30 and above 35 have declined in recent cohorts, concentrating fertility within a narrower age range (electronic supplementary material, figure S7). Lastly, the family size distribution for low-scoring males is more polarized, exhibiting both heightened childlessness rates and raised parity progression rates at higher parities (electronic supplementary material, figures S8 and S14).

These patterns are systematic, sizeable and precisely inferred by conservative hierarchical models with parameter shrinkage. They are also corroborated by raw data visualizations (see electronic supplementary material, section C). Although our data sample only covers Norway, similar patterns have been observed in neighbouring Sweden [2], indicating that there was no dysgenic *male* fertility in these parts of the Scandinavian peninsula during this period.

Two important limitations should be stressed. First, military conscription historically only covered males, so the analyses cannot assess the female CA–fertility gradient. Other studies report stronger negative CA–fertility gradients for women [1]. This also means that the ‘net’ selection on CA in Norway remains unclear. Second, our study is descriptive and does not identify the underlying drivers of the observed patterns. The CA–fertility gradient is likely related to the strong fertility gradient seen for both education and earnings in Norway and Sweden [11–13], for instance, but our study cannot disentangle causation. Stratifying analyses on, e.g. educational attainment, risks collider bias and unobserved confounding, and credible causal inference requires an instrumental variable or a reform that exclusively affects education or CA.

## Data Availability

Data and code required to estimate the statistical models are available from the Dryad Digital Repository: https://doi.org/10.5061/dryad.bnzs7h4gh [6]. The data are provided in the electronic supplementary material [14].

## Endnotes

1 Each stanine can be viewed as representing a specific range of IQ values. After rounding: a stanine of 1 (IQ < 74), 2 (74–81), 3 (81–89), 4, (89–96), 5 (96–104), 6 (104–111), 7 (111–119) and 8 (above 119).

2 An alternative measure is the correlation coefficient between observed stanine score and realized number of children across all males in cohorts that can be observed to age 50. This correlation coefficient is positive at 0.057.

3 We do not observe fertility above age 41 for the 1980 birth cohort, but this well after peak fertility and the model extrapolation (indicated in blue) seems reasonable.
